# Histopathological evaluation of *Onchocerca volvulus* nodules by microscopy and by digital image analysis for the study of macrofilaricidal drug efficacy

**DOI:** 10.3389/fmed.2023.1099926

**Published:** 2023-02-02

**Authors:** Kerstin Fischer, Bettina Dubben, Linda B. Debrah, Janina M. Kuehlwein, Arcangelo Ricchiuto, Alexander Y. Debrah, Achim Hoerauf, Gary J. Weil, Peter U. Fischer, Ute Klarmann-Schulz

**Affiliations:** ^1^Division of Infectious Diseases, Department of Medicine, School of Medicine, Washington University, St. Louis, MO, United States; ^2^Institute for Medical Microbiology, Immunology and Parasitology (IMMIP), University Hospital Bonn, Bonn, Germany; ^3^Kumasi Center for Collaborative Research (KCCR), Kumasi, Ghana; ^4^Department of Clinical Microbiology, School of Medicine and Dentistry, Kwame Nkrumah University of Science and Technology (KNUST), Kumasi, Ghana; ^5^German Center for Infection Research (DZIF), Bonn-Cologne site, Bonn, Germany; ^6^Institute of Medical Biometry, Informatics and Epidemiology (IMBIE), University Hospital Bonn, Bonn, Germany; ^7^Faculty of Allied Health Sciences, Kwame Nkrumah University of Science and Technology (KNUST), Kumasi, Ghana

**Keywords:** *Onchocerca volvulus*, histology, clinical trials, viability assessment, digital images

## Abstract

**Background:**

Novel drugs or drug combinations that kill or permanently sterilize adult *Onchocerca volvulus* worms would be very helpful for treatment and elimination of onchocerciasis. In absence of a reliable biomarker for viable adult worms, histopathological assessment of worms within onchocercal nodules is a standard method to determine macrofilaricidal activity. The goal of the present study was to determine the agreement between two independent experts in the analysis of nodule sections and to assess the value of digital imaging as a means of standardizing the analysis.

**Material and methods:**

Two expert microscopists independently assessed 605 nodules by direct microscopy. At least two sections with two different stains hematoxylin & eosin (H&E, APR immunostain) of paraffin-embedded, ethanol-fixed whole-nodule cross-sections were analyzed. After variables were identified prone to observer discrepancies, we performed a second study to compare consolidated results for 100 nodules obtained by the two readers by microscopy and by analysis of scanned, high resolution digital images (20x magnification). The last data set analyzed was a quality panel of 100 nodules that has been previously examined by microscopy, and included additional immunostains for *Wolbachia* endobacteria. These slides were digitalized, read by the two assessors and results were compared with original microscopy results.

**Results:**

The degree of agreement between assessors varied for different parameters. Agreement for female worm counts in nodules was approximately 80%, while agreement regarding female worm viability was 98%. There were no major differences observed between results obtained by microscopy or digital images. Good agreement for important parameters was also observed for the nodules of the quality panel.

**Conclusion:**

Nodule analysis by experienced microscopists was reproducible with regard to important parameters such as identification of living female worms or detection of normal embryogenesis. Assessments varied more for other parameters, and we recommend continued use of two independent readers for detailed analyzes. Analysis of scanned images provided similar results to direct microscopy. This facilitates training and comparison of nodule findings by readers in different locations. Analysis of high quality digital images that can be viewed remotely should improve the quality and availability of nodule assessments that are primary endpoints for onchocerciasis clinical trials.

## Introduction

Onchocerciasis or “river blindness” is a neglected tropical disease (NTD) caused the by the filarial parasite *Onchocerca volvulus*. This parasite causes severe eye and skin disease, and it has been linked to neurological disorders such as epilepsy and nodding syndrome (1). Onchocerciasis has been targeted for global elimination, and currently the main strategy used is community-directed treatment with the microfilaricidal drug ivermectin (CDTI) (1). While the program has made great progress with regard to reducing morbidity, transmission persists in many areas in Africa. Therefore, new drugs or drug combinations are needed that either permanently sterilize or kill the adult *O. volvulus* worms (2, 3).

Adult *O. volvulus* worm live in subcutaneous nodules, with females being stationary and males migrating between nodules. In the absence of reliable biomarkers for viable *O. volvulus* worms that can be detected in body fluids, analysis of nodules after surgical extirpation is the standard procedure for assessing the effect of drugs on adult worms during clinical trials (4–8). Also, *Wolbachia* endosymbiont densities are high in adult *O. volvulus*, and examination of nodules can be crucial for assessing the efficacy of anti-*Wolbachia* drugs in humans (9, 10). *O. volvulus* worms can live in nodules for up to 15 years. They undergo a natural aging process, and it is not always easy to differentiate age-related morphological changes from treatment-related effects. Nodule biopsies do not capture the morphology of all adult worms within the nodule. Adult worms can by assessed directly after collagenase digestion of nodules and isolation of worms or by histopathological analysis of adult worms within the nodule *in situ* (11). Both methods have advantages and disadvantages. For example, worm numbers can be more accurately determined after collagenase digestion, but host reaction to the worms inside the nodule or the presence of intra-nodular microfilariae cannot be analyzed without histopathology.

Assessment of histopathological sections can vary between different readers, but over the last 40 years specific morphological criteria for the assessment of adult worm vitality have been established. However, regulatory authorities have not agreed to the use of these criteria, and this has led to efforts to standardized histopathological assessment of *O. volvulus* nodules. One step toward this standardization is the masked assessment of histopathological sections by two independent readers. Furthermore, experimental procedures need to comply with guidelines for Good Clinical Practice (GCP) and Good Clinical Laboratory Practice (GCLP) (12–14). Unfortunately, the expertise to assess histopathological sections of *O. volvulus* nodules accurately for worm viability is vanishing, and currently only a few laboratories in Africa, Europe and the USA have proper training and knowledge to do this. This means that sometimes hundreds of histopathological sections have to be shipped from country to country or continent to continent for histopathological assessment. With advances in telemedicine it should be possible to perform histopathological worm viability assessment based on high resolution images or scans of entire nodule sections. However, the feasibility and accuracy of digital histopathological nodule assessment has not been previously assessed.

One goal of the present study was to compare conventional nodule assessment results by direct microscopy by two independent readers to determine the agreement with regard to key parameters such as number of female worms and percentages of worms that are living or supporting embryogenesis. A second goal of the study was to compare results of nodule assessments based on direct microscopy with results based on assessment of digital images from the same histopathological sections. Results of the study led to a standardized, rapid and cost effective protocol for evaluating the macrofilaricidal efficacy of new treatments for onchocerciasis.

## Materials and methods

### Nodule samples

Two different sets of nodule samples were used for the study. First-, a total of 605 nodules were available from a study which was completed in 2018 and compared the effects of multiple doses of ivermectin and ivermectin plus albendazole as part of the Death to Onchocerciasis and Lymphatic Filariasis (DOLF) project (15, 16). These treatments were not macrofilaricidal. However, even nodules from untreated subjects or subjects that were treated with drugs without macrofilaricidal activity may contain dead worms, female worms without embryogenesis or females with degenerated ovaries. A subset of these 605 nodules was relabeled to ensure masked analysis and used for digital scanning to compare results from direct microscopy with those from digital images scanned from the same nodule slides. The second set of samples comprised of an archived quality panel of sections from 100 nodules out of 43 participants from three different studies performed in Ghana that have been assessed by the late Dietrich W. Büttner (9) or by Sabine Specht (17, 18). This panel comprised a mixture of treatments [Placebo ± IVM (*n* = 28), Doxycycline 100 mg for 6 weeks (*n* = 7), Doxycycline 200 mg for 6 weeks (*n* = 3), Doxycycline 200 mg for 3 weeks + Albendazole for 3 days (*n* = 2), Doxycycline 200 mg for 4 weeks (*n* = 1), Doxycycline 200 mg for 3 weeks (*n* = 1), Albendazole for 3 days (*n* = 1)] and of different operation time points (6, 20, or 27 months after treatment onset). In addition to the standard stains described below these nodules were also stained using an antibody against the *Wolbachia* surface protein to detect *Wolbachia* endosymbionts as described previously (19) (Table 1). Doxycycline 200 or 100 mg for 6 weeks leads to *Wolbachia* depletion (after 6 months) (9), and sterility of adult female worms (17, 19–21) and for 200 mg additional macrofilaricidal effects (after 20–27 months) could be shown (9). Doxycycline 200 mg for 4 weeks also leads to *Wolbachia* depletion after 6 months (18) and sterility of adult female worms after 20–27 months (9).

**TABLE 1:** Overview of nodule samples and stains analyzed for the present study.

Number of nodules	Project	Stains (slides analyzed)	Comparison	Equipment used for reading	Transfer of slides or images
605	DOLF (15)	H&E, APR, Gomori’s (3)	Analog results from 2 readers	Standard laboratory microscope	Airmail (DHL)
100	DOLF (15)	H&E, APR (2)	Analog vs. digital results	Olympus vs. 120 scanner, standard laboratory microscope	Cloud, https://wustl.account.box.com
100	Quality panel (10, 17, 18)	H&E, APR, WSP (3)	Digital results from 2 readers vs. analog results reference panel	Zeiss scanner, Zeiss laboratory microscope	Cloud, https://www.hrz.uni-bonn.de/en/all-services/data-storage-fileservices/sciebo-hochschulcloud.nrw?set_language=en

### Histology

The histopathological procedures were performed as previously described (15). Briefly, nodules were fixed in 80% ethanol and sections were stained with Mayer’s hematoxylin & eosin (H&E, Merck, Darmstadt, Germany), anti-aspartic protease sera (APR) for viability (22) and the Gomori’s iron stain for age determination. Histopathological procedures were performed at IMMIP, University Hospital in Bonn, Germany.

### Evaluation of nodules by direct microscopy

Each slide had two consecutive nodule sections. Two or three nodule sections (stains) per nodule were evaluated by two independent readers (Tables 2, 3), who were blinded regarding patient treatment history and the other reader’s results. Microscopical assessment of nodule sections was done using a standard light microscope (Zeiss Primostar, Carl Zeiss, Jena, Germany) equipped with Primo Plan-ACROMAT objectives with 4X, 10X, 40X, and 100X magnification and WF 10 × 18 oculars. One reader (BD) was located at the Institute for Medical Microbiology, Immunology and Parasitology (IMMIP), University Hospital Bonn, Germany and the other reader (KF) was located at Washington University in St. Louis, MO, USA. After the first nodule reading by microscopy was completed in Germany, nodule sections were shipped to the US for the second reading.

**TABLE 2:** Comparison of the agreement on the nodule level of two independent readers assessing nodules from a clinical trial using microscopy.

	Reader 1	Reader 2	Number of nodules with agreement	% agreement per nodule	Inter-rater reliability [Cohen’s Kappa k; (*P*-value)]
No. of worms total	1,692	1,735	481	79.5	0.744 (*p* < 0.001)
No. of females (% total no. of worms)	1,470 (86.9)	1,495 (86.2)	475	78.5	0.725 (*p* < 0.001)
No. of males (% total no. of worms)	222 (13.1)	240 (13.8)	564	93.2	0.856 (*p* < 0.001)
No. of worms alive (% total no. of worms)	838 (49.5)	867 (50)	538	88.9	0.854 (*p* < 0.001)
No. of worms dead (% total no. of worms)	854 (50.5)	868 (50)	500	82.6	0.772 (*p* < 0.001)
No. of nodules with alive females (% total no. of nodules)	369 (61.0)	375 (62.0)	595	98.3	0.965 (*p* < 0.001)
No. of nodules with dead females (% total no. of nodules)	414 (68.4)	390 (64.5)	567	93.7	0.859 (*p* < 0.001)
No. nodules with free Mf (% no. of nodules)	34 (9.3)	32 (8.7)	365	99.5	0.967 (*p* < 0.001)
No. of nodules with embryogenesis judgeable (%)	337 (91.8)	342 (93.2)	358	97.5	0.823 (*p* < 0.001)
No. of nodules with uterus empty (%)	55 (16.4)	69 (20.6)	321	95.8	0.862 (*p* < 0.001)
No. of nodules with oocytes only (%)	170 (50.7)	156 (46.6)	321	95.8	0.917 (*p* < 0.001)
No. of nodules with degenerated embryos only (%)	39 (11.6)	38 (11.3)	330	98.5	0.927 (*p* < 0.001)
No. of nodules with normal embryogenesis (%)	70 (20.9)	72 (21.5)	333	99.4	0.982 (*p* < 0.001)
No. of nodules with normal morulae	42	42	333	99.4	0.973 (*p* < 0.001)
No. of nodules with normal coiled	46	46	335	100	1.0 (*p* < 0.001)
No. of nodules with normal stretched	62	65	332	99.1	0.971 (*p* < 0.001)

Out of 605 nodules that were assessed, 367 nodules had sufficient details to assess free Mf in the nodule, and embryogenesis could be judged by both readers in 335 nodules.

**TABLE 3:** Patient level comparison of direct microscopy nodule results from two readers.

	Reader 1	Reader 2	Number of patients	% agreement per patient	Interrater reliability [Cohen’s Kappa κ; (*P*-value)]
Patients with live female worms (%)	179 (83.3)	179 (83.3)	215	100	1 (*p* < 0.001)
Patients with females and judgeable embryogenesis (%)	174 (81)	176 (82)	179	98.9	0.745 (*p* < 0.001)
Patients with females and normal embryogenesis (%)	60 (28)	61 (28.3)	174	99.4	0.987 (*p* < 0.001)
Patients with females and degenerated embryogenesis only (%)	28 (13)	28 (13)	174	97.7	0.915 (*p* < 0.001)

The histology technicians were specifically trained to assess the morphology of *O. volvulus* worms and have assessed thousands of nodule sections before. They had extensive experience in microscopy and histopathological procedures and were able to differentiate technical artifacts from morphological changes. The readers classified the morphology of *O. volvulus* according to previously published criteria (4, 11, 23, 24). Briefly, dead worms were differentiated from living worms by the presence of disintegrated nuclei and the absence of APR labeling (Supplementary Figures 1, 2). Male worms were identified by their size, the presence of a single genital tube and the narrow annulations of the cuticle. The larger females have paired genital tubes mostly filled with developing embryos. The state of embryogenesis within the living female worm was judged by the presence of intact morula-, coiled-, pretzel- or stretched-stage embryos within the two uterus branches (Supplementary Figures 3, 4). All nodules were part of treatment trials and the effect of treatments varied by drug or drug combination. To facilitate comparisons, slides were read in a unified orientation relative to the barcode label. Unless clearly identifiable as males, dead worms were recorded as dead females. Results from each nodule were recorded on a case report form (CRF) that included number and sex of worms, presence of microfilariae in the nodule and viability and embryogenesis of each female worm in the nodule. This CRF was modified from previously published forms (11).

We compared the two readers’ nodule evaluations focused on the agreement between number of identified worms (females, males), viability and assessment of embryogenesis (stage of embryos, integrity of embryos) (Table 2). In addition, combined nodule results from individual patients were compared (Table 3). The nodule assessments of both readers were compared using statistical procedures described below. After the assessment comparison was completed, readers met in person or virtually to resolve discrepancies and consolidate results.

### Comparison of results obtained by direct microscopy with those obtained from scanned images

This evaluation was performed with 100 nodules (two slides per nodule, H&E stain, APR immunostain). Gomori’s iron stain was not included in the scans, because the worms were of similar age. After the assessment was completed, the readers met virtually to resolve any discordance. The final result from the digital image reading was then compared to the final results from the microscopic assessment (Table 4).

**TABLE 4:** Comparison of worm numbers obtained by direct microscopy and analysis of digital images from 100 nodules from a clinical trial.

	Microscopy	Digital image analysis	% agreement per nodule/worms	Interrater reliability [Cohen’s Kappa κ; (*P*-value)]
No of nodules with living females (%)	60 (60%)	61 (61%)	99%	0.979 (*p* < 0.001)
Total no of worms	255	209	56%	0.413 (*p* < 0.001)
No of females (%)	220 (86.3%)	176 (84.2%)	57%	0.425 (*p* < 0.001)
No of dead females (%)	122 (55.5)	86 (48.9%)	65%	0.499 (*p* < 0.001)
No of living females (%)	98 (44.5%)	90 (51.1%)	83%	0.752 (*p* < 0.001)

Nodules were assessed by two readers for both microscopy and digital image analyzes, and any discrepancies between the two readers were resolved prior to comparison of results obtained by the two methods.

### Additional evaluation of scanned images

In order to evaluate the performance of the two readers using digital images we used a quality panel that was previously analyzed by microscopy by the late D.W. Büttner (24) or by S. Specht (17, 18). In those studies sections were also stained with WSP, to assess presence and abundance of *Wolbachia* (Table 5) (19). This quality panel was also used for teaching purposes and D.W. Büttner, who did the microscopic evaluation had also access to metadata (for example a couple of nodules were from children and contained only young worms).

**TABLE 5:** Comparison of direct microscopy and digital analysis of 100 nodules from a clinical trial.

	Microscopy	Digital image analysis	No. of Nodule	% agreement per nodule	Interrater reliability [Cohen’s Kappa κ; (*P*-value)]
Free Mf present	7	8	61	60/61 (98.4%)	0.924 (*p* < 0.001)
Embryogenesis in females judgeable (%)	53 (88.3%)	57 (95%)	60	54/60 (90%)	0.355 (*p* = 0.002)
Females that contain oocytes only (%)	31 (77.5%)	32 (80%)	40	31/40 (77.5%)	0.328 (*P* = 0.37)
Females with normal embryogenesis present (%)	7 (17.5%)	7 (17.5%)	40	40/40 (100%)	1.0 (*p* < 0.001)
Females with degenerated embryos only present (%)	4 (1%)	8 (2%)	40	32/40 (80%)	0.231 (*p* = 0.114)

Nodules were assessed by two readers for both microscopy and digital image analyzes, and any discrepancies between the two readers were resolved prior to comparison of results obtained by the two methods.

### Generation and analysis of digital images

We used two different scanning methods to ensure that results from the digital image analysis are not limited to a single slide scanner. Pilot studies were performed to evaluate the impact of key parameters (magnification, scanning speed, and resolution/quality) on outcomes. Using an Olympus scanner (Olympus vs. 120 Brightfield Slide Scanning System, Tokyo, Japan) it took 3–5 min at 20X magnification to scan an entire nodule section, but up to 10–15 min at 40X magnification. Scanning speed and quality was dependent on the size of the nodule, potential wrinkles of the section, homogeneity of thickness of the section and potential bubbles that occurred under the coverslip. While scanning time is not an important issue for a single slide, it becomes important if a study examines 500 nodules with 2 or 3 sections each which could take up to 10.5 days non-stop scanning at 40X. We found that a 20X resulted in sufficient resolution to examine single nuclei or a small cluster of *Wolbachia* endobacteria and that this could be accomplished with a feasible scanning speed. Scanned images were stored on an external hard drive and uploaded to a cloud server. The average size of a scan was 9.5 GB, and images were assessed using the free viewing software OlyVia 2.19 (Olympus). For evaluation of the quality panel, slides were scanned at 20X magnification using a Zeiss scanner (AxioScan, Carl Zeiss, Jena, Germany). Those images were saved as jpg files (average 38 MB per slide), and analyzed using open domain picture viewing software (i.e., Windows Photo Viewer, Microsoft, Seattle, WA, USA). Results were recorded using one CRF per nodule.

### Statistical analysis

Data were recorded on a paper CRF and then entered into REDCap (Research Electronic Data Capture, Vanderbilt University, Nashville, TN, USA) (25, 26) using double-data entry. Results were downloaded into Excel (Microsoft Corp., Redmond, WA, USA) and compared with SAS version 9.4 (SAS Institute Inc., Cary, NC, USA) using standard procedures. Descriptive statistics were performed using SPSS 27.0 (IBM, Armonk, NY, USA). Cohen’s Kappa index was calculated to assess inter-reader reproducibility. The Kappa index was grouped into 6 classes including no agreement (*k* < 0), slight (*k* = 0–0.20), fair (*k* = 0.21–0.40), moderate (k = 0.41–0.60), substantial (*k* = 0.61–0.80), and almost perfect agreement (*k* = 0.81–1) using standard thresholds (27).

## Results

### Direct microscopy assessment of nodules by two independent readers

In a first step, we analyzed the agreement between two readers, using their original data from a previous, clinical trial, before any discrepancies were resolved (15). We looked at the overall agreement of the total number of worms, before venturing onto the nodule level or comparing the different parameters on the number of nodules. Agreement between the readers ranged between substantial (*k* = 0.61– 0.80) and nearly perfect (*k* = 0.81–1) for different parameters. The readers mostly disagreed on the number of alive or dead females, and that affected the numbers of total worms. In total Reader 1 detected 1.7% fewer female worms, and there was no significant difference between the total numbers of female worms detected in the 605 nodules (Table 2). Because in the nodule the female worms are coiled up, it is not possible to accurately determine their number. Although the overall estimates of worm numbers were similar, the agreement of detected number of females per nodule was only 78.5% but this includes dead and alive females.

More important than consistent numbers of worms is the correct differentiation between living and dead worms. The agreement between the readers on the number of nodules with alive or dead females was nearly perfect (98.3 and 93.7%) respectively. This was also true for the number of nodules with microfilariae, where the agreement was 99.5%. Another important parameter was the assessment of embryogenesis in living female worms. Embryos are judged as being normal or degenerated, but it is in some cases embryogenesis cannot be judged at all. For example, this happens when the uterus is empty.

While there was slight disagreement regarding the number of nodules with judgeable embryogenesis, agreement regarding the stage of present embryos was near perfect or perfect. For all embryogenesis-related parameters, the total agreement ranged between 80 and 100% and the agreement per nodule ranged between 96 and 100%.

While analysis at the level of the nodule level is scientifically interesting and has greater statistical power with regard to accuracy of nodule reading, one also should consider a patient-level analysis for evaluation of clinical trial results. Patient level results had stronger levels of agreement than nodule or worm level results [98–100% agreement regarding the presence of living female worms, female worms with intact embryogenesis or with degenerated embryogenesis (Table 3)]. This shows that although there are some differences between readers, with regard to variables essential to the outcome of clinical trials the agreement between two trained readers is very high.

### Comparison of microscopical and digital assessments of nodules from a clinical trial

**FIGURE 1:**
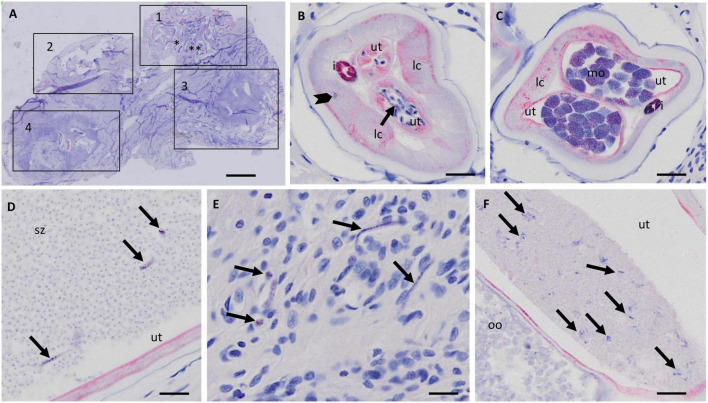
Resolution of nodule sections scanned with the Olympus vs. 120 scanner at 20X magnification. **(A)** Overview of a scanned nodule (APR stain) with at least four female worms. The numbered squares indicate coiled females. **(B)** Zoomed-in cross section of female 1 with normal stretched Mf in the uterus (arrow). A macronucleus in the lateral cord is visible (arrowhead). **(C)** Zoomed-in cross section of the same female as in panel **(B)** [marked with two asterisks in panel **(A)**] with normal morulae. **(D)** Uterus with numerous spermatozoa and normal stretched microfilaria (arrows). **(E)** Zoomed-in nodule tissue around female 1 with free Mf (arrows). **(F)** Zoomed-in cross-section of uterus of female 3 with degenerated Mf (arrow) in one uterus branch and normal oocytes in the other uterus branch. Ut, uterus; lc, lateral chord; i, intestine; mo, morulae; sz, spermatozoa; oo, oocytes. Scale bar 2 mm in **(A)**, 25 μm in panels **(B–F)**.

**FIGURE 2:**
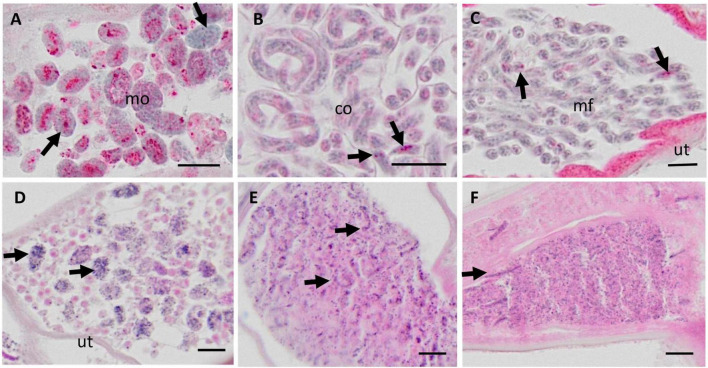
Micrographs of various embryonic stages in the uterus of live female *Onchocerca volvulus*, magnified from whole nodule images scanned with the Olympus vs. 120 scanner at 20X magnification. **(A–C)** APR stained nodules for viability assessment; **(D–F)** H&E stained nodule sections. **(A)** Normal morulae with APR positive lysosomes (red stain). Note the nuclei with condensed chromatin (arrows). **(B)** Normal coiled embryos in an eggshell and with few APR positive lysosomes (arrows). **(C)** Normal stretched microfilariae without eggshell and few APR positive lysosomes (arrows) **(D)** Degenerated morulae (arrows) with small, disintegrating nuclei. **(E)** Degenerated coiled and stretched embryos (arrows) with small, disintegrating nuclei. **(F)** Many degenerated microfilariae with small, disintegrating nuclei, and single stretched intact microfilariae (arrow). Mo, morulae; co, coiled microfilariae; mf, microfilariae; ut, uterus. Scale bar 10 μm.

In a first step the suitability of scanned nodule sections for the detailed assessment of worm morphology was evaluated. It was concluded that scanning at 20-fold magnification allowed to identify and judge sufficient details for a comprehensive nodule assessment (Figure 1). For example scanning of an entire nodule with a diameter of about 2 cm, allowed assessment of intact and degenerated Mf in the uterus as well as detection of intrauterine spermatozoa and morula-stage embryos (Figures 1, 2). This qualitative evaluation of scanned cross-sections of entire nodules showed that sufficient resolution can be achieved to evaluate also smaller structures such as integrity of nuclei, cross-sections of intra-nodular Mf and developing embryos (Figure 2).

After deciding on parameters for scanning, slides from 100 stained nodules from the DOLF clinical trial were randomly selected, and scanned for digital assessment. After resolving any discordances, the results from the microscopy and digital reading were compared (Table 4). The agreement between microscopy and digital nodule assessment with regard to the identification of nodules with living females was almost perfect with 99%. There was also substantial agreement with regard to the identification of living female worms at the level of individual worms but only a moderate agreement with regard to the total number of females and the number of dead females. In general digital nodule reading resulted in a slightly lower number of females compared to direct microscopy.

Because there was a difference in the assessment of the total number of female worms between direct microscopy and digital reading, we focused on the assessment of fertility at the level of the nodule rather than on fertility of individual worms. There was an almost perfect agreement with regard to the detection of free, intra-nodular Mf and for detection of nodules with normal embryogenesis (Table 5). Although the overall assessments for the ability to judge the embryogenesis in females, the presence of females that only contained oocytes, and the presence of females that contained only degenerated embryos without intact embryos by microscopy and digital reading led to similar results, the actual agreement per sample was only fair.

### Direct microscopy and digital nodule assessment of a quality panel by independent readers

**FIGURE 3:**
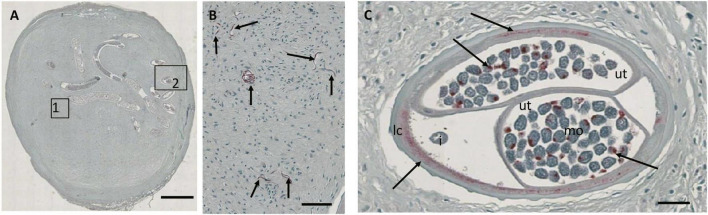
JPG image from a Zeiss Axioscop scan (20X magnification) of a nodule section that was stained for *Wolbachia* surface protein (Wsp, stained red). **(A)** Overview of the nodule with a single female. **(B)** Zoomed-in area 1 showing multiple free Mf positive for Wsp (arrows). **(C)** Zoomed-in cross-section showing *Wolbachia* stained in the lateral chord and normal morulae (arrows). Ut, uterus; i, intestine; lc, lateral chord; mo, morulae. Scale bar 2 mm in **(A)**, 25 μm in panels **(B,C)**.

In order to better link current nodule reading outcome to historical published data, we used a quality panel of 100 nodules that has been assessed by direct microscopy performed by the late D.W. Büttner (11) or by S. Specht. Results of Büttner’s assessments were published long ago, but they are still considered the standard for nodule assessment. Paper records of previous nodules readings using microscopy were entered into RedCap for analysis and slides were scanned (see Table 1) for masked reading by the two independent readers. In contrast to the other sample sets, these nodules were also examined for the presence and density of *Wolbachia* endobacteria. A scanned nodule section stained for *Wolbachia* surface protein allowed detection of *Wolbachia* in Mf, the lateral chord of female worms, and in morula stage embryos (Figure 3).

Table 6 shows that a high level of agreement between readers 1 and 2 and between the readers and the reference was achieved. The lowest percentage of agreement, ranging between 63.6 and 73.5%, was observed for the number of female worms at the nodule level. However, for most other parameters agreement was nearly perfect with exception of *Wolbachia* density (which also had substantial agreement). Although the analysis of complex morphological structures by direct microscopy or by high resolution digital images can result in some discrepancies, the evaluation of a historical quality panel showed that analysis of digital images led to similar results concerning critical variables such as identification of living and fertile female *O. volvulus* worms.

**TABLE 6:** Comparison of the results of digital image analysis by two different reader of a quality panel that was previously assessed by microscopy of 100 *Onchocerca* nodules.

	Reader 1 vs. reference		Reader 2 vs. reference		Reader 1 vs. Reader 2	
	% agreement per nodule/Worm	Interrater reliability [Cohen’s Kappa κ; (*P*-value)]	% agreement per nodule/Worm	Interrater reliability [Cohen’s Kappa κ; (*P*-value)]	% agreement per nodule/Worm	Interrater reliability [Cohen’s Kappa κ; (*P*-value)]
No. nodules with Mf	87/94 (92.6%)	0.808 (*p* < 0.001)	84/92 (91.3%)	0.786 (*p* < 0.001)	89/94 (94.7%)	0.854 (*p* < 0.001)
No. of females	72/98 (73.5%)	0.59 (*p* < 0.001)	63/97 (64.9%)	0.477 (*p* < 0.001)	63/99 (63.6%)	0.457 (*p* < 0.001)
No. of males	83/98 (84.7%)	0.723 (*p* < 0.001)	84/95 (89.5%)	0.81 (*p* < 0.001)	91/97 (93.8%)	0.887 (*p* < 0.001)
No. of alive female worms	87/97 (89.7%)	0.676 (*p* < 0.001)	73/95 (76.8%)	0.633 (*p* < 0.001)	69/97 (71.1%)	0.568 (*p* < 0.001)
No. with embryogenesis judgeable	87/89 (97.8%)	na*	88/89 (98.9%)	na*na	88/89 (98.9%)	na*
No. of nodules Uterus NOT empty	84/86 (97.7%)	0.491 (*p* < 0.001)	85/87 (97.7%)	0.739 (*p* < 0.001)	83/87 (95.4%)	0.32 (*p* < 0.001)
No. with oocytes only	68/82 (82.9%)	0.642 (*p* < 0.001)	73/81 (90.1%)	0.785 (*p* < 0.001)	71/79 (89.9%)	0.79 (*p* < 0.001)
No. with normal embryogenesis	76/82 (92.7%)	0.853 (*p* < 0.001)	77/82 (93.9%)	0.878 (*p* < 0.001)	77/81 (95.1%)	0.899 (*p* < 0.001)
No. with degenerated embryos only	74/82 (90.2%)	0.502 (*p* < 0.001)	72/82 (87.8%)	0.478 (*p* < 0.001)	71/81 (87.7%)	0.437 (*p* < 0.001)
No. with *Wolbachia*	84/92 (91.3%)	0.553 (*p* < 0.001)	81/88 (92%)	0.548 (*p* < 0.001)	85/88 (96.6%)	0.781 (*p* < 0.001)
Few vs. many	63/78 (80.8%)	0.424 (*p* < 0.001)	58/75 (77.3%)	0.368 (*p* < 0.001)	68/79 (86.1%)	0.649 (*p* < 0.001)

For each nodule at least four different, consecutive sections stained by three different stains (H&E, APR stain, Gomori’s stain or WSP-1 stain) were analyzed. *n.a = Cohen’s kappa could not be interpreted due to the small number of nodules assessed as having alive female worms but no judgeable embryogenesis (*n* = 1 for reader 1 and reference, *n* = 0 for reader 2).

## Discussion

This study has shown that although there was limited disagreement between independent readers with regard to some variables, such as number of female worms in a nodule, there was good agreement with regard to critical variables such as female worm viability and fertility. These results have confirmed that morphological analysis of nodules is a reliable tool to assess worm viability. Furthermore, the study showed that high resolution digital images of entire nodule sections can be used to identify critical morphological characters for reliable assessment of nodules after treatment trials.

Trials to develop new treatments for onchocerciasis that require nodulectomy and assessment of nodules for worm viability have been scarce in the last two decades (9, 10, 15, 17–19, 28). Therefore, the number of histopathology experts/invertebrate pathologists with experience in the assessment of worm viability is decreasing. These experts rely mostly on reference observations published many years ago, before the widespread community drug treatment with ivermectin (11, 29, 30). Therefore, in many areas worm populations are often older, female worms may be temporarily sterilized and newly acquired female worms are less common (23, 24). It is easier to detect treatment effects in young and healthy worm populations with ongoing transmission, and the assessment of older worm populations requires additional expertise. For this reason and in order to better standardize the nodule assessment, two independent readers should be used. In our study the agreement between the two readers with regard to worm numbers and embryogenesis was substantial or almost perfect (Table 2). However, it has to be noted that estimates of worm numbers are not always easy, because female worms are coiled up and sometimes intertwined. Female worms of the same age are more difficult to differentiate and older females often have a wider distribution within the nodule and may be intertwined with other females. Also the determination of number of dead female worms has some inconsistency, because a dead female may be partially resorbed, calcified or difficult to differentiate from a dead male worm. Given these problems, the good agreement between both independent readers is astonishing.

It has to be noted that worm assessment in nodules not only provides information about worm numbers, viability and reproductive status, but also about other morphological features that may be linked to drug treatment. For example morphological features that indirectly affect worm viability include recruitment of macrophages and other immune cells toward the worm’s cuticle, the presence of neoplasms and the absence of *Wolbachia* endobacteria (19, 31, 32).

We observed 99% agreement with regard to identification of nodules with living females by microscopy and by digital image analysis. Given the difficulties to determine worm numbers, it is not surprising that the agreement between both methods for this parameter was somewhat lower. However with regard to the identification of intra-nodular Mf and females with normal embryogenesis the agreement between microscopy and digital image analysis was 98.4 and 100%, respectively. Since identifying living females and females with normal embryogenesis is most important for determining the outcome of clinical trials, it can be concluded that digital image analysis as described in the present study can replace direct microscopy. A recent meta-analysis of 25 studies concluded equivalent performance of digital pathology in comparison with light microscopy for routine diagnosis of medical conditions (33). Digital image analysis is especially important in Africa and other areas of the world where well-trained pathologists are scarce (34, 35). Slide scanners are more and more available in core facilities also in Africa, have similar energy requirements as microscopes, and scanning can be performed by any laboratory technician after a short training session. Therefore, slide scanning should be feasible in most histopathology laboratories in developing countries.

*Onchocerca volvulus* nodules are usually 1–3 cm in diameter; analysis of an entire nodule cross-section needs to detect morphological structures that are only one μm in size, such as a small cluster of 2 or 3 *Wolbachia* endobacteria. Our study describes a scanning algorithm that achieved satisfying resolution with two different slide scanners while being practical in regard to scanning time per slide, and an acceptable file size for storage and data transfer. Analysis of a quality panel that was previously analyzed by microscopy confirmed that the agreement between two independent readers of digital images was similar to the agreement of each reader with the quality panel. It has to be noted that in contrast to the readers of the digital images, the original assessor of the quality panel by microscopy was not masked with regard to treatment group, age of the patient and number of nodules from the same patient. Therefore, the high level of agreement in this study is remarkable.

Apart from the local availability of trained nodule readers, there are also logistic advantages in favor of the employment of digital images analysis. For example for the reading of slides by microscopy for a single clinical trial that analyzed 617 nodules, 19 slide boxes with 1,851 slides needed to be shipped from the University Bonn, in Germany to Washington University in St. Louis, MO, USA (15). Broken slides needed to be re-stained and after the second reading the slides needed to be sent back to Germany for archiving per protocol. Other advantages are that digital images can be more easily linked to digital case report forms and that digital images can be analyzed by two or even more readers at the same time. The digital method also allows the two assessors to meet virtually and discuss the results (provided internet speed is sufficient), especially in the event of discrepancies in the evaluation, down to the smallest detail, e.g., down to the individual section of a worm. In the case of microscopic evaluation, the two readers would otherwise have to sit at a microscope with 2 oculars in order to be able to talk about the same section. Making annotations on individual details in the images is also quite easy with the help of basic image processing programs and helpful for the later agreement and clarification of questions between the two assessors. These advantages facilitate analysis and shorten the time of clinical trials. Disadvantages of the use of digital images are the fact that is takes additional time and computer storage to generate the images and that slide scanners are costly. Since the scanning process is mostly automated, the hands-on time is limited and should not be a main factor. During the last years the costs for computer storage and for slide scanners are decreasing and giving the total costs of clinical trials these expenses should be negligible.

The use of high resolution digital nodule images is a first step toward automated image analysis using artificial intelligence. Digital pathology is a fast evolving field and already in 2017 the US Food and Drug Administration allowed marketing of the first whole slide imaging system for regulatory purposes (36).

## Conclusion

We have shown that although there may be some disagreements between independent nodule readers with regard to worm number estimates, there is an almost perfect agreement between readers with regard to the identification of living female worms and female worms with normal embryogenesis. There is also a strong agreement between the slide reading by microscopy and by digital image analysis. The use of digital image analysis of nodule sections in clinical trials for onchocerciasis can be recommended, and two independent histopathology experts should assess the digital images in parallel to increase robustness of results.

## Data availability statement

The raw data supporting the conclusions of this article will be made available by the authors, without undue reservation.

## Ethics statement

The clinical trial was registered at International Standard Randomized Controlled Trial Number (https://doi.org/10.1186/ISRCTN50035143) and was approved by the Ethical Committees in Ghana, Germany and the USA as mentioned before (15). The use of archived, de-identified nodule samples for teaching and research purposes does not constitute human subjects research.

## Author contributions

UK-S and PF conceived the study. LD and AD provided the essential materials for the study. KF and BD collected the experimental data for the study. UK-S, JK, AR, and KF performed the data analysis. KF and PF wrote the first draft of the manuscript. AH and GW edited the manuscript. All authors have read, commented on, and approved the last version of the manuscript.

## Supplementary material

Supplementary Figure 1 **(A)** APR stained nodule with four worm nests. The numbered squares show where the nest are located. Square 1 and 3 are nests with alive worms, recognizable because the worms are APR positive (red). **(B)** Image of a partially resorbed worm (arrows). Only worm remnants and large cells are visible (arrowhead). Located in square 2 * of **(A)**. **(C)** One cross-section of an old alive female worm with coiled empty uteri. Located between square 3 and 4 (^**^). **(D)** One cross-section of a dead female. The worm is APR negative and therefore not stained red. Section is located between square 3 and 4 (^**^). Large macrophage-like cells are in the vicinity of the cuticle (arrowhead). **(E)** H&E stained nodule with one female worm and one male worm (star). **(F)** Alive male worm with spermatozoa in the vas deferens. Notice the nuclei (arrowhead) which indicate that the male is alive, and the serrated cuticula (arrow) as well as surrounding neutrophils. **(G)** APR stained male (APR positive, stained red) with surrounding neutrophils. Notice the intact nuclei (arrowheads) in the lateral chord, and the spermatids in the testis. Ut, uterus; lc, lateral chord; i, intestine, vd, vas deferens; t, testis; sz, spermatozoa; sp, spermatids.

Supplementary Figure 2 **(A)** H&E stained nodule with at least 3 dead worms. **(B)** Dead worm sections (arrowheads) located in square 3 with one male worm section (arrow) which could be alive. **(C)** Dead worm section (arrow) surrounded by giant cell (stars). Located in square 1. **(D)** APR stained nodule, same as in panel **(A)**. One worm section is stained red, indicating that the worm is still alive. **(E)** Higher magnification of worm sections found in square 3. APR positive worm section (arrow) surrounded by APR negative worm sections (arrowheads). **(F)** Higher magnification of worm section found in square 1. Now one can see that this is an old, but alive male worm with positive APR stain. The serrated cuticule (arrows) identifies this section as male worm.

Supplementary Figure 3 Micrographs showing cross-sections of adult *Onchocerca volvulus*, magnified from whole nodule images scanned with the Olympus vs. 120 scanner at 20X magnification. **(C,D)** APR stained nodules for viability assessment; **(A,B)** H&E stained nodule sections. **(A)** Anterior part of an *O. volvulus* female that has the same diameter as a male worm. The female has a well-developed lateral chord. The uterus is paired, and one branch contains stretched microfilariae (arrow). **(B,C)** Two *O. volvulus* males. Males are smaller in diameter and shorter in length compared to females. Males have well developed muscle cells and a single genital tube. The testis contains spermatocytes in panel **(B)** and more mature spermatids in panel **(C)**. The cuticle of the male in panel **(C)** shows typical deep striations (arrow) in contrast to the cuticle of females, where the striations are further apart (arrowhead). **(D)** Midbody region of a female with intact early embryonic stages in the uterus. The small morulae are positive for *Ov-APR*. **(E)** Midbody region of another female with degenerated embryos, that are not *Ov*-APR positive. Lc, lateral, chord; mc, median chord; i, intestine; mc, muscle cells; ut, uterus; t, testis; sc, spermatocytes; sp, spermatids; oo, oocytes. Scale bar 25 μm.

Supplementary Figure 4 Micrographs showing cross-sections of adult *Onchocerca volvulus* with varying viability, magnified from whole nodule images scanned with the Olympus vs. 120 scanner at 20X magnification. **(A–C,F)** APR stained nodules for viability assessment; **(D,E)** H&E stained nodule sections. **(A)** Living female worm with degenerated stretched microfilaria in one uterus string (arrow). **(B)** Living female worm with large pleomorphic neoplasm in the pseudocoelomic cavity. **(C–F)** Dead female worms, APR negative and no intact nuclei in the worm recognizable. The worms are mostly calcified. **(C)** Dead female worm with calcified organ structures. APR negative. **(D)** Almost resorbed dead female worm with circular arrangement of collagen (arrows). **(E)** Remnants of a dead calcified female. **(F)** Dead female with disintegrated organs. Lc, lateral, chord; i, intestine; ut, uterus; ne, neoplasm. Scale bar, 25 μm.

## References

[1] World Health Organization [WHO]. Progress report on the elimination of human onchocerciasis, 2019-2020. *Wkly Epidemiol Rec.* (2020) 95:545–56.

[2] Fischer PU King CL Jacobson JA Weil GJ. Potential value of triple drug therapy with ivermectin, diethylcarbamazine, and albendazole (IDA) to accelerate elimination of lymphatic filariasis and onchocerciasis in Africa. *PLoS Negl Trop Dis.* (2017) 11:e0005163. 10.1371/journal.pntd.0005163 28056015 PMC5215784

[3] Ngwewondo A Scandale I Specht S. Onchocerciasis drug development: from preclinical models to humans. *Parasitol Res.* (2021) 120:3939–64. 10.1007/s00436-021-07307-4 34642800 PMC8599318

[4] Albiez EJ Walter G Kaiser A Ranque P Newland HS White AT Histological examination of onchocercomata after therapy with ivermectin. *Trop Med Parasitol.* (1988) 39:93–9. 3175472

[5] Awadzi K Hero M Opoku NO Addy ET Buttner DW Ginger CD. The chemotherapy of onchocerciasis XVIII. Aspects of treatment with suramin. *Trop Med Parasitol.* (1995) 46:19–26. 7631123

[6] Awadzi K Hero M Opoku O Buttner DW Gilles HM. The chemotherapy of onchocerciasis. XV. Studies with albendazole. *Trop Med Parasitol.* (1991) 42:356–60. 1796233

[7] Buttner DW. The significance of morphological studies on macrofilariae from onchocerciasis patients for the evaluation of control measures. *Trop Med Parasitol.* (1985) 36(Suppl. 1):2–4. 4039843

[8] Duke BO Zea-Flores G Munoz B. The embryogenesis of onchocerca volvulus over the first year after a single dose of ivermectin. *Trop Med Parasitol.* (1991) 42:175–80. 1801140

[9] Hoerauf A Specht S Buttner M Pfarr K Mand S Fimmers R Wolbachia endobacteria depletion by doxycycline as antifilarial therapy has macrofilaricidal activity in onchocerciasis: a randomized placebo-controlled study. *Med Microbiol Immunol.* (2008) 197:295–311. 10.1007/s00430-007-0062-1 17999080 PMC2668626

[10] Hoerauf A Specht S Marfo-Debrekyei Y Buttner M Debrah AY Mand S Efficacy of 5-week doxycycline treatment on adult onchocerca volvulus. *Parasitol Res.* (2009) 104:437–47. 10.1007/s00436-008-1217-8 18850111

[11] Buttner DW Albiez EJ von Essen J Erichsen J. Histological examination of adult onchocerca volvulus and comparison with the collagenase technique. *Trop Med Parasitol.* (1988) 39(Suppl. 4):390–417. 2852393

[12] Food and Drug Administration [FDA]. Regulations: *Good Clinical Practice and Clinical Trials*. (2021). Available online at: https://www.fda.gov/science-research/clinical-trials-and-human-subject-protection/regulations-good-clinical-practice-and-clinical-trials

[13] Ezzelle J Rodriguez-Chavez IR Darden JM Stirewalt M Kunwar N Hitchcock R Guidelines on good clinical laboratory practice: bridging operations between research and clinical research laboratories. *J Pharm Biomed Anal.* (2008) 46:18–29. 10.1016/j.jpba.2007.10.010 18037599 PMC2213906

[14] Vijayananthan A Nawawi O. The importance of good clinical practice guidelines and its role in clinical trials. *Biomed Imaging Interv J.* (2008) 4:e5. 10.2349/biij.4.1.e5 21614316 PMC3097692

[15] Batsa Debrah L Klarmann-Schulz U Osei-Mensah J Dubben B Fischer K Mubarik Y Comparison of repeated doses of ivermectin versus ivermectin plus albendazole for treatment of onchocerciasis - a randomized open-label clinical trial. *Clin Infect Dis.* (2019) 71:933–43. 10.1093/cid/ciz889 31536624 PMC7428389

[16] Weil GJ Fischer PU *The DOLF Project: Death to Onchocerciasis and Lymphatic Filariasis St. Louis, MO*. (2022). Available online at: https://dolfproject.wustl.edu/10.1371/journal.pntd.0013953 PMC12867263 41632749

[17] Debrah AY Specht S Klarmann-Schulz U Batsa L Mand S Marfo-Debrekyei Y Doxycycline leads to sterility and enhanced killing of female onchocerca volvulus worms in an area with persistent microfilaridermia after repeated ivermectin treatment: a randomized, placebo-controlled, double-blind trial. *Clin Infect Dis.* (2015) 61:517–26. 10.1093/cid/civ363 25948064 PMC4518165

[18] Klarmann-Schulz U Specht S Debrah AY Batsa L Ayisi-Boateng NK Osei-Mensah J Comparison of doxycycline, minocycline, doxycycline plus albendazole and albendazole alone in their efficacy against onchocerciasis in a randomized, open-label, pilot trial. *PLoS Negl Trop Dis.* (2017) 11:e0005156. 10.1371/journal.pntd.0005156 28056021 PMC5215804

[19] Hoerauf A Mand S Volkmann L Buttner M Marfo-Debrekyei Y Taylor M Doxycycline in the treatment of human onchocerciasis: kinetics of wolbachia endobacteria reduction and of inhibition of embryogenesis in female onchocerca worms. *Microbes Infect.* (2003) 5:261–73. 10.1016/S1286-4579(03)00026-1 12706439

[20] Hoerauf A Volkmann L Hamelmann C Adjei O Autenrieth IB Fleischer B Endosymbiotic bacteria in worms as targets for a novel chemotherapy in filariasis. *Lancet.* (2000) 355:1242–3. 10.1016/S0140-6736(00)02095-X 10770311

[21] Hoerauf A Mand S Adjei O Fleischer B Büttner DW. Depletion of wolbachia endobacteria in onchocerca volvulus by doxycycline and microfilaridermia after ivermectin treatment. *Lancet.* (2001) 357:1415–6. 10.1016/S0140-6736(00)04581-5 11356444

[22] Jolodar A Fischer P Buttner DW Miller DJ Schmetz C Brattig NW. Onchocerca volvulus: expression and immunolocalization of a nematode cathepsin D-like lysosomal aspartic protease. *Exp Parasitol.* (2004) 107:145–56. 10.1016/j.exppara.2004.06.006 15363940

[23] Specht S Brattig N Buttner M Buttner DW. Criteria for the differentiation between young and old onchocerca volvulus filariae. *Parasitol Res.* (2009) 105:1531–8. 10.1007/s00436-009-1588-5 19784672 PMC2764059

[24] Specht S Hoerauf A Adjei O Debrah A Buttner DW. Newly acquired onchocerca volvulus filariae after doxycycline treatment. *Parasitol Res.* (2009) 106:23–31. 10.1007/s00436-009-1624-5 19756742 PMC2780640

[25] Harris PA Taylor R Thielke R Payne J Gonzalez N Conde JG. Research electronic data capture (REDCap)–a metadata-driven methodology and workflow process for providing translational research informatics support. *J Biomed Inform.* (2009) 42:377–81. 10.1016/j.jbi.2008.08.010 18929686 PMC2700030

[26] Harris PA Taylor R Minor BL Elliott V Fernandez M O’Neal L The REDCap consortium: building an international community of software platform partners. *J Biomed Inform.* (2019) 95:103208. 10.1016/j.jbi.2019.103208 31078660 PMC7254481

[27] Landis JR Koch GG. The measurement of observer agreement for categorical data. *Biometrics.* (1977) 33:159–74. 10.2307/2529310 843571

[28] Awadzi K Opoku NO Attah SK Lazdins-Helds J Kuesel AC. A randomized, single-ascending-dose, ivermectin-controlled, double-blind study of moxidectin in onchocerca volvulus infection. *PLoS Negl Trop Dis.* (2014) 8:e2953. 10.1371/journal.pntd.0002953 24968000 PMC4072596

[29] Duke BO Zea-Flores G Gannon RT. On the reproductive activity of the female onchocerca volvulus. *Trop Med Parasitol.* (1990) 41:387–402. 2075383

[30] Striebel HP. Proposed form to evaluate some histological aspects of macrofilarial morphology, its age dependent alterations and drug related changes in nodules of onchocerca volvulus and O. gibsoni. *Trop Med Parasitol.* (1988) 39(Suppl. 4):367–89. 3227241

[31] Brattig NW Hoerauf A Fischer PU Liebau E Bandi C Debrah A Immunohistological studies on neoplasms of female and male onchocerca volvulus: filarial origin and absence of wolbachia from tumor cells. *Parasitology.* (2010) 137:841–54. 10.1017/S0031182009992010 20199697 PMC2925449

[32] Duke BO Marty AM Peett DL Gardo J Pion SD Kamgno J Neoplastic change in onchocerca volvulus and its relation to ivermectin treatment. *Parasitology.* (2002) 125:431–44. 10.1017/S0031182002002305 12458827

[33] Azam AS Miligy IM Kimani PK Maqbool H Hewitt K Rajpoot NM Diagnostic concordance and discordance in digital pathology: a systematic review and meta-analysis. *J Clin Pathol.* (2021) 74:448–55. 10.1136/jclinpath-2020-206764 32934103 PMC8223673

[34] Mremi A Bentzer NK McHome B Mlay J Blaakaer J Rasch V The role of telepathology in diagnosis of pre-malignant and malignant cervical lesions: implementation at a tertiary hospital in Northern Tanzania. *PLoS One.* (2022) 17:e0266649. 10.1371/journal.pone.0266649 35421156 PMC9009664

[35] Silas OA Abdulkareem F Novo JE Zheng Y Nannini DR Gursel DB Telepathology in nigeria for global health collaboration. *Ann Glob Health.* (2022) 88:81. 10.5334/aogh.3673 36196362 PMC9479662

[36] Food and Drug Administration [FDA]. *FDA Allows Marketing of First Whole Slide Imaging System for Digital Pathology*. (2017). Available online at: https://www.fda.gov/news-events/press-announcements/fda-allows-marketing-first-whole-slide-imaging-system-digital-pathology

