# Supplementary figures and images for: Histopathological evaluation of Onchocerca volvulus nodules by microscopy and by digital image analysis for the study of macrofilaricidal drug efficacy
Source: Front Med (Lausanne). 2023 Feb 2;10:1099926. doi: 10.3389/fmed.2023.1099926 (PMC9932808; doi:10.3389/fmed.2023.1099926)

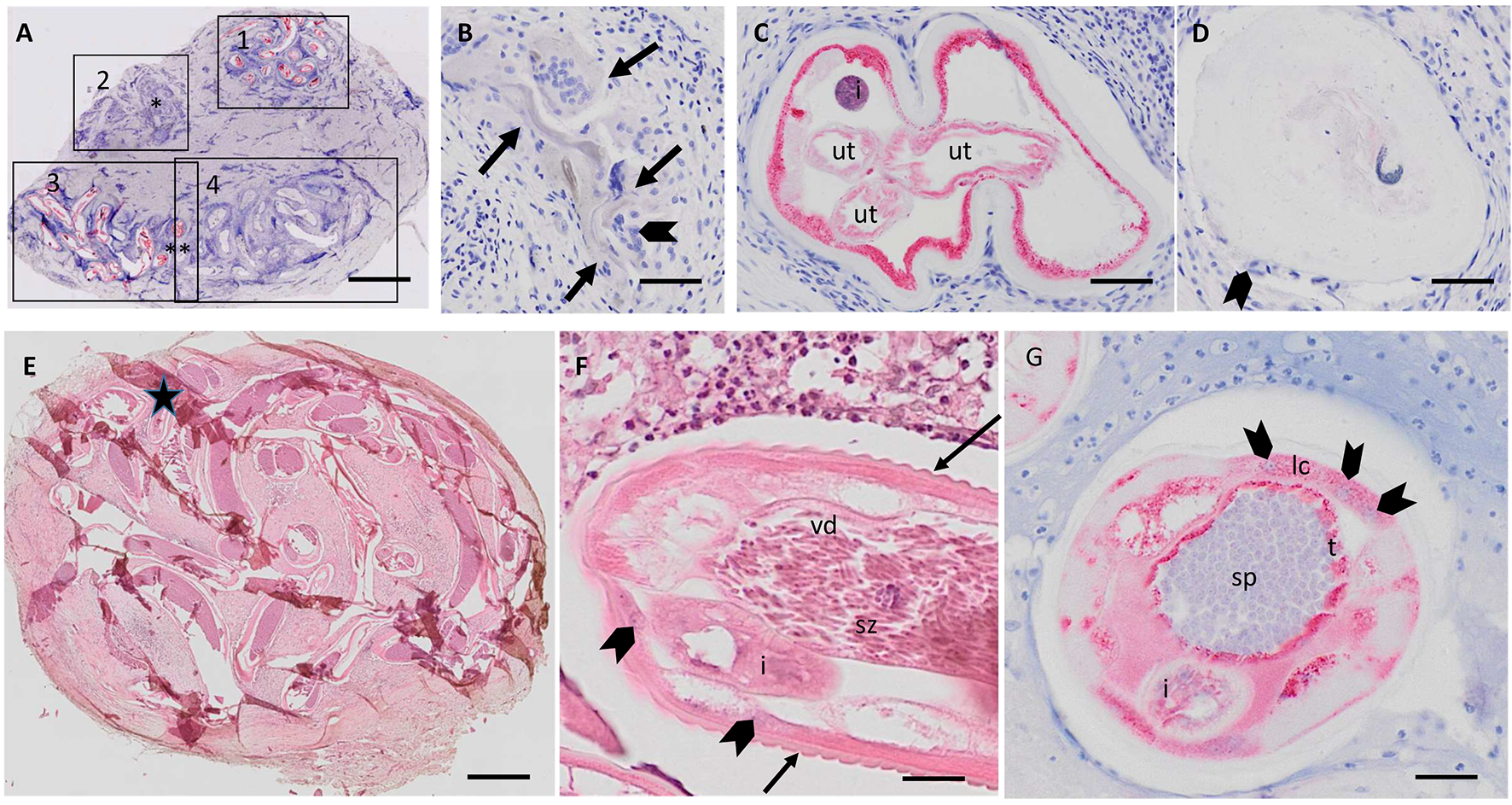

Supplement: Supplementary Figure 1 — (A) APR stained nodule with four worm nests. The numbered squares show where the nest are located. Square 1 and 3 are nests with alive worms, recognizable because the worms are APR positive (red). (B) Image of a partially resorbed worm (arrows). Only worm remnants and large cells are visible (arrowhead). Located in square 2 * of (A). (C) One cross-section of an old alive female worm with coiled empty uteri. Located between square 3 and 4 (**). (D) One cross-section of a dead female. The worm is APR negative and therefore not stained red. Section is located between square 3 and 4 (**). Large macrophage-like cells are in the vicinity of the cuticle (arrowhead). (E) H&E stained nodule with one female worm and one male worm (star). (F) Alive male worm with spermatozoa in the vas deferens. Notice the nuclei (arrowhead) which indicate that the male is alive, and the serrated cuticula (arrow) as well as surrounding neutrophils. (G) APR stained male (APR positive, stained red) with surrounding neutrophils. Notice the intact nuclei (arrowheads) in the lateral chord, and the spermatids in the testis. Ut, uterus; lc, lateral chord; i, intestine, vd, vas deferens; t, testis; sz, spermatozoa; sp, spermatids. [file Image_1.TIF]

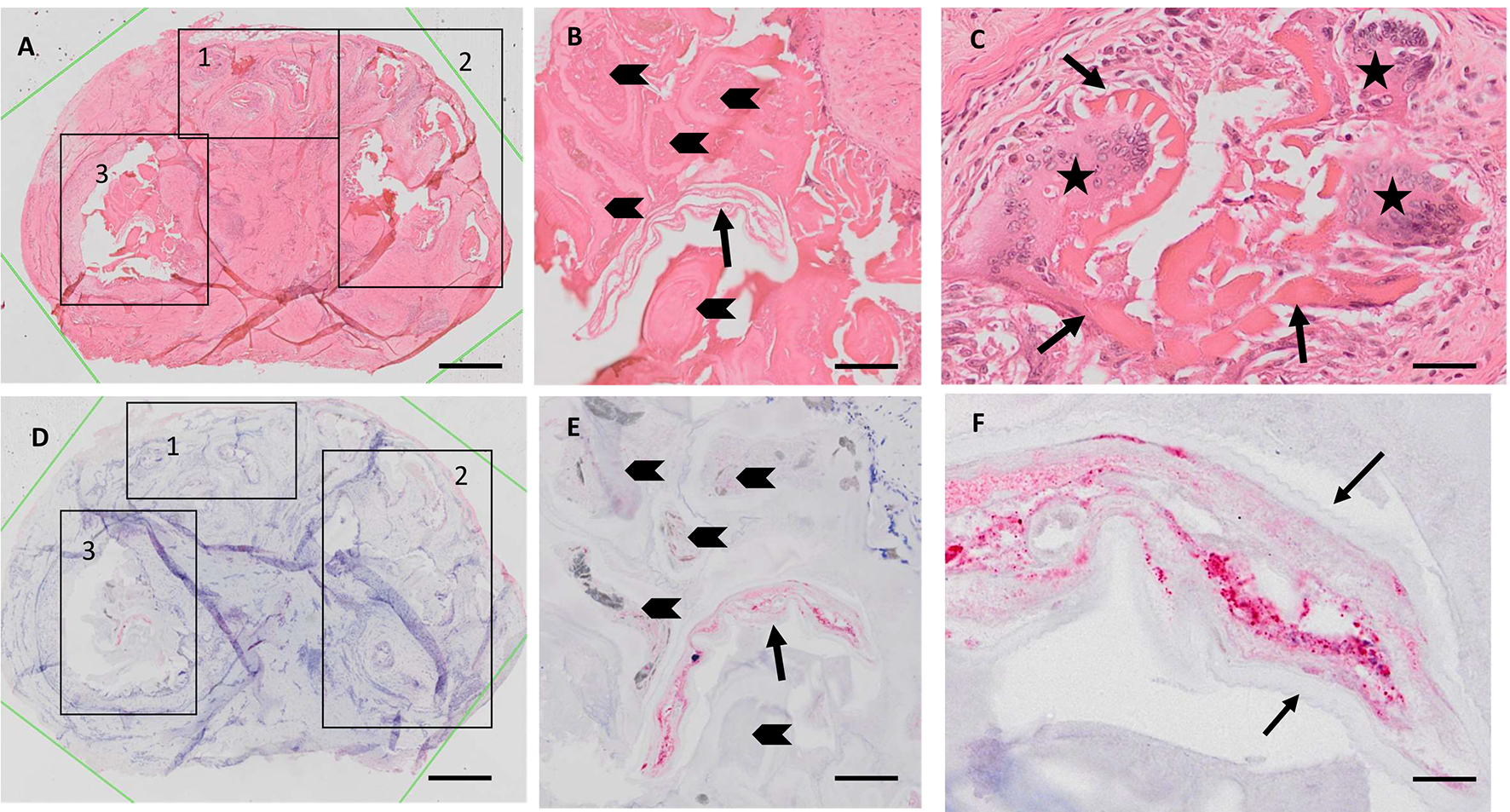

Supplement: Supplementary Figure 2 — (A) H&E stained nodule with at least 3 dead worms. (B) Dead worm sections (arrowheads) located in square 3 with one male worm section (arrow) which could be alive. (C) Dead worm section (arrow) surrounded by giant cell (stars). Located in square 1. (D) APR stained nodule, same as in panel (A). One worm section is stained red, indicating that the worm is still alive. (E) Higher magnification of worm sections found in square 3. APR positive worm section (arrow) surrounded by APR negative worm sections (arrowheads). (F) Higher magnification of worm section found in square 1. Now one can see that this is an old, but alive male worm with positive APR stain. The serrated cuticule (arrows) identifies this section as male worm. [file Image_2.TIF]

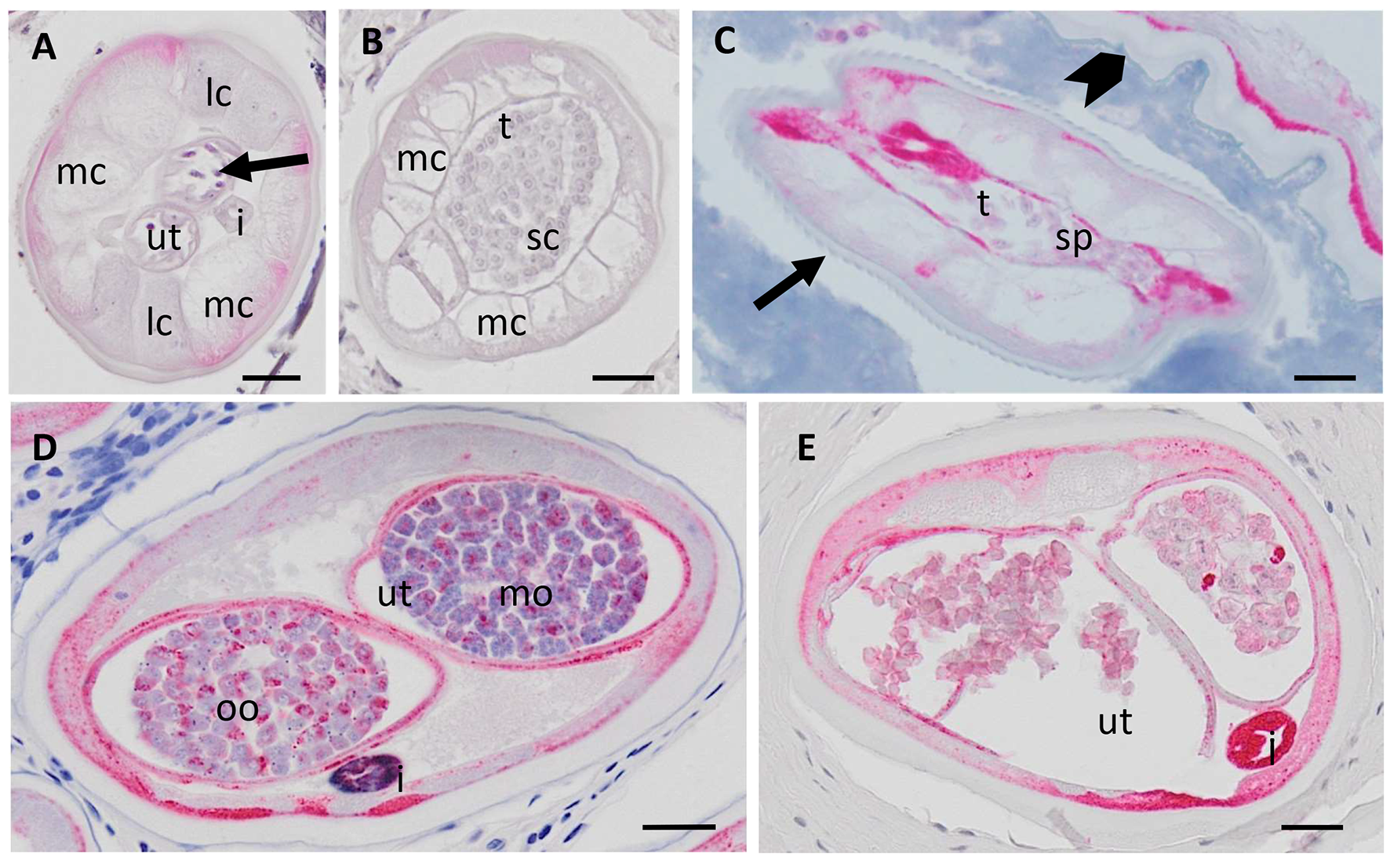

Supplement: Supplementary Figure 3 — Micrographs showing cross-sections of adult Onchocerca volvulus, magnified from whole nodule images scanned with the Olympus vs. 120 scanner at 20X magnification. (C,D) APR stained nodules for viability assessment; (A,B) H&E stained nodule sections. (A) Anterior part of an O. volvulus female that has the same diameter as a male worm. The female has a well-developed lateral chord. The uterus is paired, and one branch contains stretched microfilariae (arrow). (B,C) Two O. volvulus males. Males are smaller in diameter and shorter in length compared to females. Males have well developed muscle cells and a single genital tube. The testis contains spermatocytes in panel (B) and more mature spermatids in panel (C). The cuticle of the male in panel (C) shows typical deep striations (arrow) in contrast to the cuticle of females, where the striations are further apart (arrowhead). (D) Midbody region of a female with intact early embryonic stages in the uterus. The small morulae are positive for Ov-APR. (E) Midbody region of another female with degenerated embryos, that are not Ov-APR positive. Lc, lateral, chord; mc, median chord; i, intestine; mc, muscle cells; ut, uterus; t, testis; sc, spermatocytes; sp, spermatids; oo, oocytes. Scale bar 25 μm. [file Image_3.TIF]

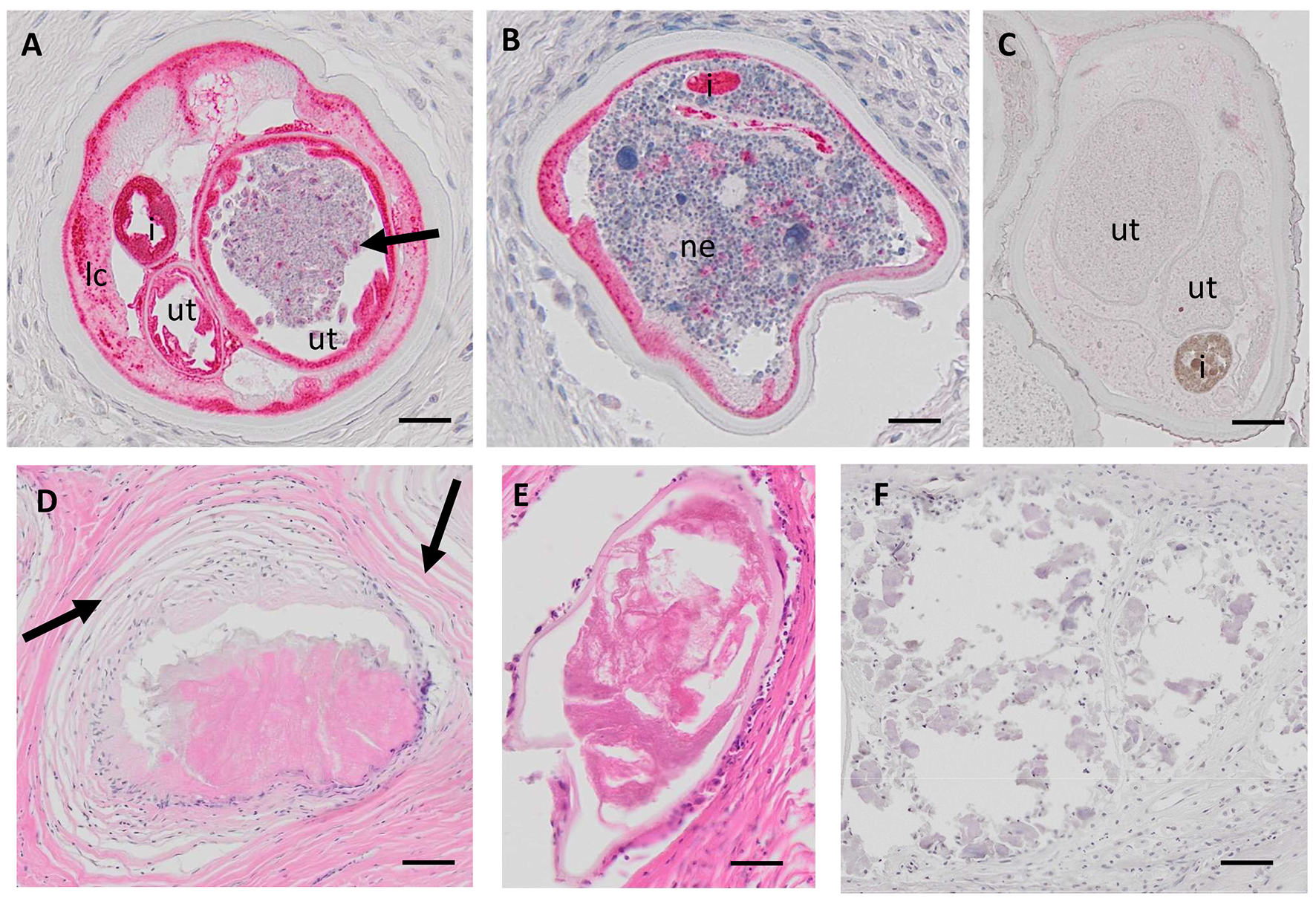

Supplement: Supplementary Figure 4 — Micrographs showing cross-sections of adult Onchocerca volvulus with varying viability, magnified from whole nodule images scanned with the Olympus vs. 120 scanner at 20X magnification. (A–C,F) APR stained nodules for viability assessment; (D,E) H&E stained nodule sections. (A) Living female worm with degenerated stretched microfilaria in one uterus string (arrow). (B) Living female worm with large pleomorphic neoplasm in the pseudocoelomic cavity. (C–F) Dead female worms, APR negative and no intact nuclei in the worm recognizable. The worms are mostly calcified. (C) Dead female worm with calcified organ structures. APR negative. (D) Almost resorbed dead female worm with circular arrangement of collagen (arrows). (E) Remnants of a dead calcified female. (F) Dead female with disintegrated organs. Lc, lateral, chord; i, intestine; ut, uterus; ne, neoplasm. Scale bar, 25 μm. [file Image_4.TIF]
